# Supplementary figures and images for: Elucidating the Prognostic and Therapeutic Implications of Insulin Resistance Genes in Breast Cancer: A Machine Learning-Powered Analysis
Source: Biology (Basel). 2025 May 13;14(5):539. doi: 10.3390/biology14050539 (PMC12109394; doi:10.3390/biology14050539)

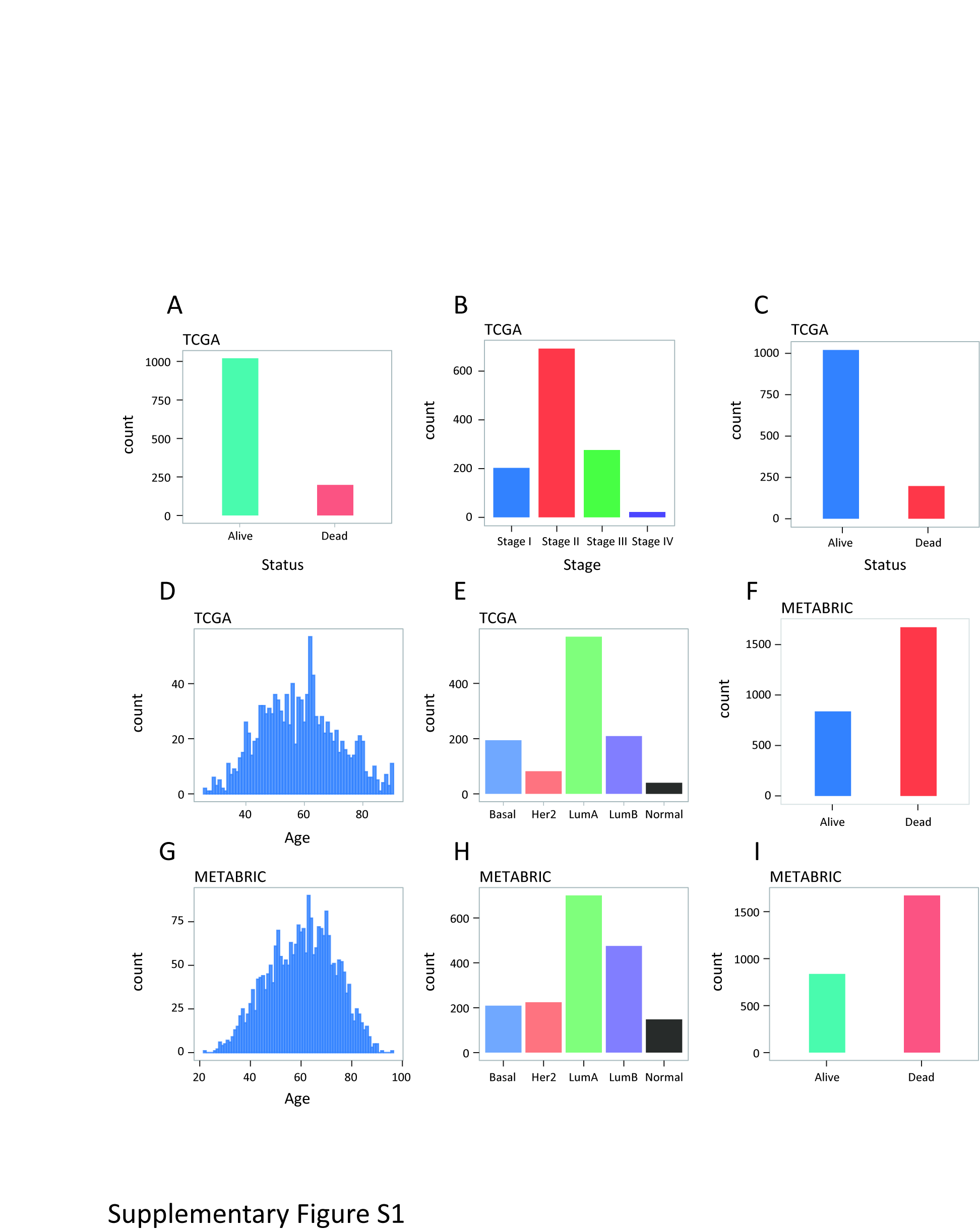

Supplement: Supplementary file 1 [file biology-14-00539-s001.zip › Supplementary Figure S1.tif]

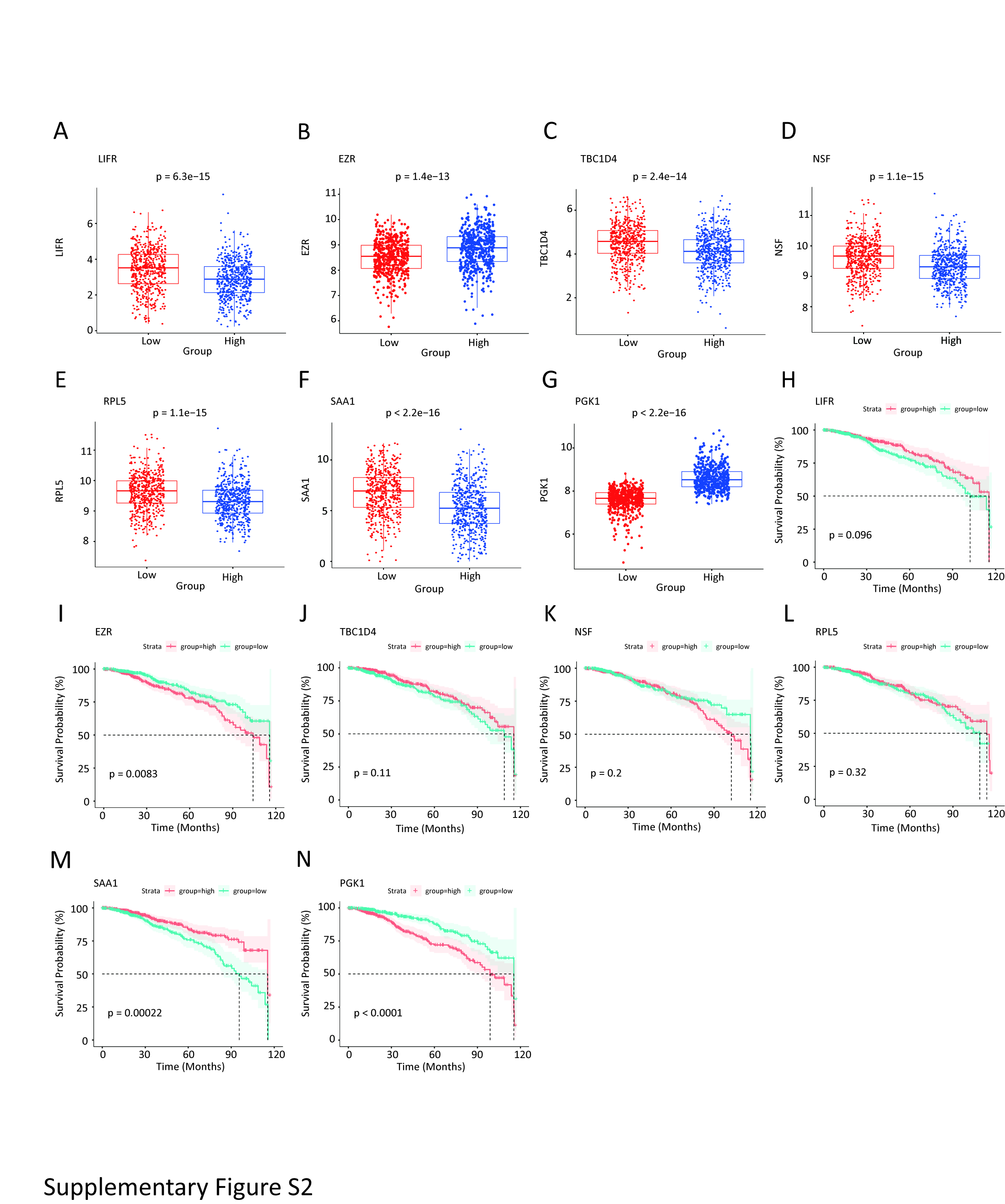

Supplement: Supplementary file 1 [file biology-14-00539-s001.zip › Supplementary Figure S2.tif]

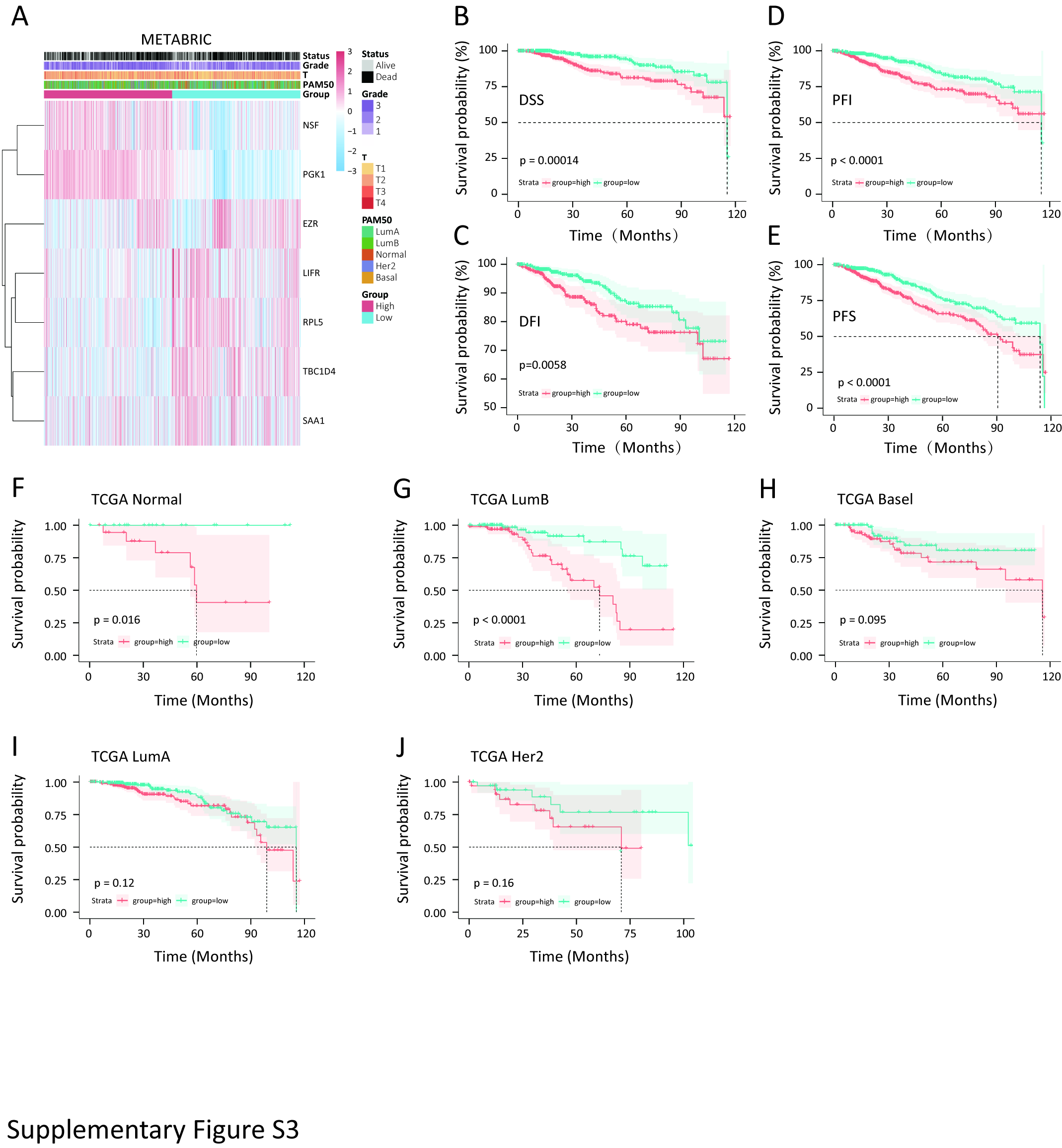

Supplement: Supplementary file 1 [file biology-14-00539-s001.zip › Supplementary Figure S3.tif]

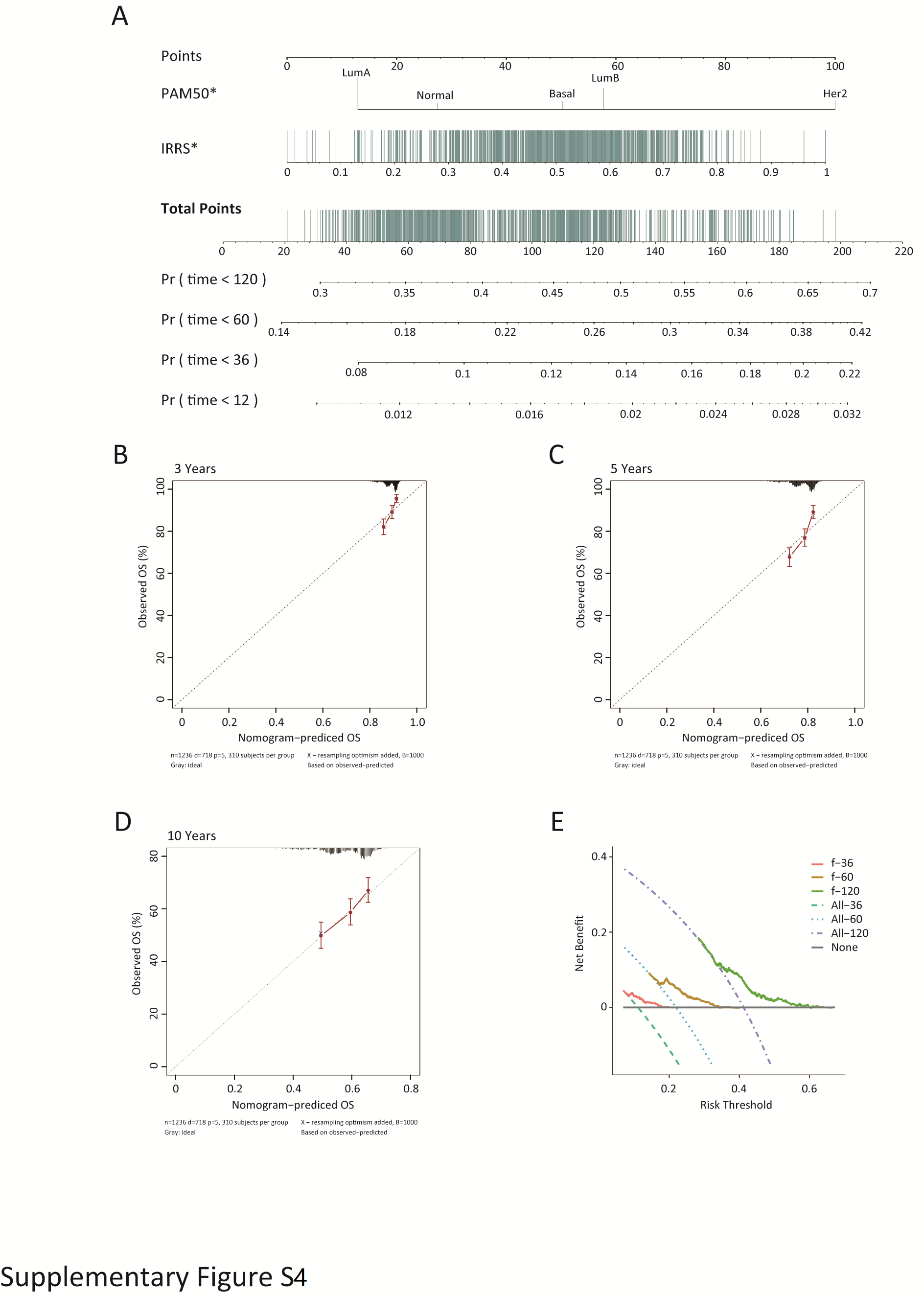

Supplement: Supplementary file 1 [file biology-14-00539-s001.zip › Supplementary Figure S4.tif]

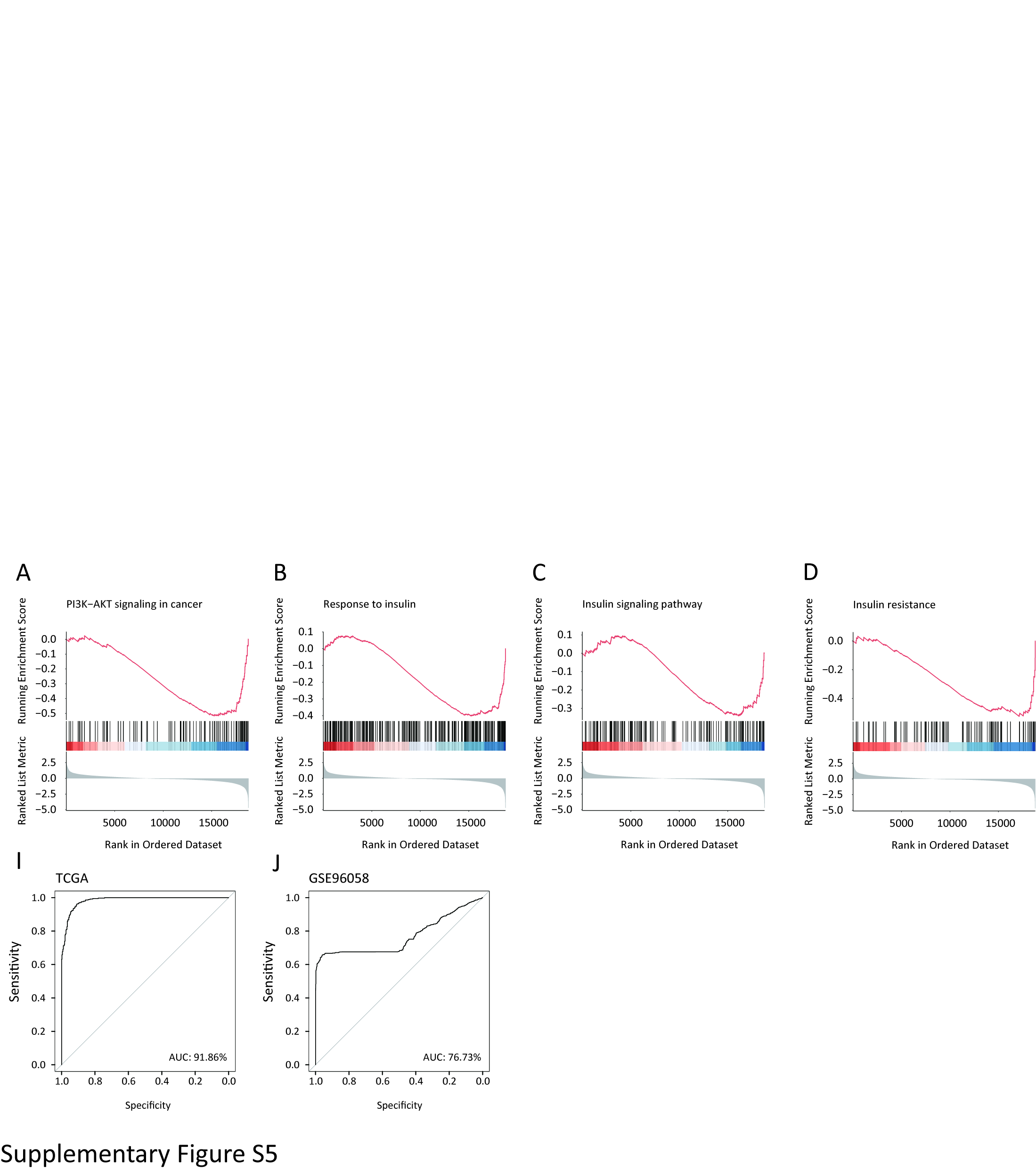

Supplement: Supplementary file 1 [file biology-14-00539-s001.zip › Supplementary Figure S5.tif]
